# Translational Study of Copy Number Variations in Schizophrenia

**DOI:** 10.3390/ijms23010457

**Published:** 2021-12-31

**Authors:** Min-Chih Cheng, Wei-Hsien Chien, Yu-Shu Huang, Ting-Hsuan Fang, Chia-Hsiang Chen

**Affiliations:** 1 Department of Psychiatry, Yuli Branch, Taipei Veterans General Hospital, Hualien 981, Taiwan; uncle055@yahoo.com; 2 Department of Occupational Therapy, College of Medicine, Fu Jen Catholic University, New Taipei City 242, Taiwan; wchien58@gmail.com; 3 Department of Psychiatry, Chang Gung Memorial Hospital-Linkou, Taoyuan 333, Taiwan; yushuhuang1212@gmail.com; 4 School of Medicine, College of Medicine, Chang Gung University, Taoyuan 333, Taiwan; genie.cgu@gmail.com; 5 Department and Institute of Biomedical Sciences, Chang Gung University, Taoyuan 333, Taiwan

**Keywords:** schizophrenia, copy number variations, real-time quantitative PCR, chromosomal microarray analysis

## Abstract

Rare copy number variations (CNVs) are part of the genetics of schizophrenia; they are highly heterogeneous and personalized. The CNV Analysis Group of the Psychiatric Genomic Consortium (PGC) conducted a large-scale analysis and discovered that recurrent CNVs at eight genetic loci were pathogenic to schizophrenia, including 1q21.1, 2p16.3 (NRXN1), 3q29, 7q11.23, 15q13.3, distal 16p11.2, proximal 16p11.2, and 22q11.2. We adopted a two-stage strategy to translate this knowledge into clinical psychiatric practice. As a screening test, we first developed a real-time quantitative PCR (RT-qPCR) panel that simultaneously detected these pathogenic CNVs. Then, we tested the utility of this screening panel by investigating a sample of 557 patients with schizophrenia. Chromosomal microarray analysis (CMA) was used to confirm positive cases from the screening test. We detected and confirmed thirteen patients who carried CNVs at these hot loci, including two patients at 1q21.1, one patient at 7q11.2, three patients at 15q13.3, two patients at 16p11.2, and five patients at 22q11.2. The detection rate in this sample was 2.3%, and the concordance rate between the RT-qPCR test panel and CMA was 100%. Our results suggest that a two-stage approach is cost-effective and reliable in achieving etiological diagnosis for some patients with schizophrenia and improving the understanding of schizophrenia genetics.

## 1. Introduction

Schizophrenia is a complex mental disorder affecting approximately 1% of the general population worldwide. Genetic factors play a major role in its etiology [1]. The genetic landscapes of schizophrenia include common variants with minimal clinical effects, rare variations with high penetrance and large clinical impact, and epigenomic dysregulation [1,2]. Copy number variations (CNVs) are genetic variations resulting from the rearrangements of DNA segments. Accumulating studies show that rare CNVs (including microduplications and microdeletions) contribute a significant part to the genetic etiology of schizophrenia [3,4]. Identifying the genetic bases of psychiatric disorders can aid in achieving the etiological diagnosis of patients with psychiatric disorders and may help elucidate the pathophysiology of psychiatric disorders and hopefully improve psychiatric treatment in the future [5,6].

For example, to study pathophysiology and neuropsychiatric manifestations of the rare pathogenic CNVs associated with psychiatric disorders, two working groups were formed by the Enhancing NeuroImaging Genetics through Meta-Analysis (ENIGMA) consortium formed: the ENIGMA 22q11.2 Deletion Syndrome Working Group (22q-ENIGMA WG) and the ENIGMA-CNV Working Group (ENIGMA-CNV WG) [7]. These two working groups have reported the effects of several rare pathogenic CNVs, including 22q11.2 [7], 16p11.2 distal [8], 15q11.2 [9], and 1q21.2 [10], on brain structure, cognition, and neuropsychiatric symptoms, which has increased our understanding of the genetic etiology and neurobiology of schizophrenia and related psychiatric disorders.

However, pathogenic CNVs associated with schizophrenia are highly heterogeneous and personalized. In addition, they account for only a small portion of the genetic deficits of schizophrenia. The CNV Analysis Group of the Psychiatric Genomics Consortium (PGC) recently conducted a large-scale genome-wide CNV analysis in 21,094 schizophrenia patients and 20,227 controls. They confirmed a global enrichment of CNV burden in schizophrenia patients compared to controls. Furthermore, they found that recurrent CNVs at eight genetic loci significantly increased the risk of schizophrenia, with the odds ratios ranging from 3.8 to infinity [11]. Their findings indicate that these eight loci are hot regions of recurrent pathogenic CNVs in schizophrenia patients.

Chromosomal microarray analysis (CMA) is a molecular genetic test that can accurately detect the location, type, and size of CNVs throughout the genome. CMA significantly increases the diagnostic yield in developmental disorders with unknown etiology and high genetic loading [12]; hence, it has been recommended as the first-tier genetic test for patients with developmental disabilities or congenital abnormalities such as children affected with global developmental delay, autism spectrum disorders, or multiple congenital abnormalities [13,14,15]. Several researchers also proposed that CMA should be used as a first-tier genetic test for schizophrenia in clinical settings, as it can help identify the pathogenic CNVs and guide the clinical management of schizophrenia patients [16,17,18,19]. CMA is an array-based comparative genomic hybridization technology that needs special instruments and well-trained personnel to conduct the experiments. CMA helps identify unknown pathogenic CNVs throughout the genome, but it is not cost-effective to screen known pathogenic CNVs associated with well-documented diseases in clinical laboratories.

Real-time quantitative PCR (RT-qPCR) is a sensitive method with suitable reproducibility and flexibility to validate locus-specific CNVs across the genome [20,21]. We have used this method as a complementary assay to validate the CNVs identified in our previous CNV studies [22,23]. To facilitate translating the knowledge gained from the CNV Analysis Group of the Psychiatric Genomics Consortium (PGC) [11] to clinical psychiatric practice, we conducted a two-stage study. The specific aim of the first-stage experiment was to develop an RT-qPCR test panel that could simultaneously detect CNVs at eight genetic loci reported by PGC [11] as a screening test. The specific aim of the second-stage experiment was to test the utility of the RT-qPCR CNV screening test panel developed from the first-stage experiment by screening a sample of patients diagnosed with schizophrenia. CMA was used to verify positive cases from the screening study. We hypothesized that this two-stage approach would be a more cost-effective and efficient method for detecting known CNVs in clinical settings.

## 2. Results

### 2.1. Development and Optimization of RT-qPCR Test

For each target CNV locus, we designed an optimal pair of primers that could specifically detect the dose changes in the gene encompassed by the target CNV. These eight primer pairs can be used for RT-qPCR under the same conditions simultaneously, so they were used as the screening panel to detect CNV at eight target loci. The information of target genes, primer sequences, the size of each amplicon, the slope of the standard curve, and the regression coefficient of each standard curve at eight target loci are listed in Table 1.

### 2.2. Screening of Patients with Schizophrenia

**Figure 1:**
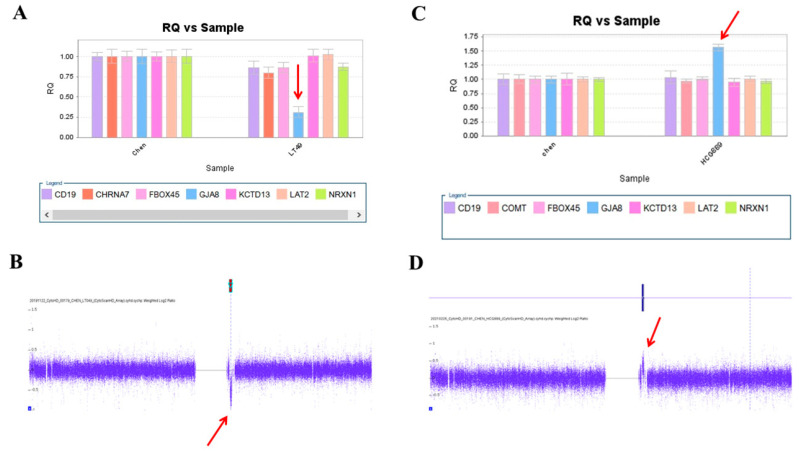
A microdeletion and a microduplication at 1q21.2 were detected in this study. (**A**) A single copy of GJA8 was detected in the patient LT48 by real-time quantitative PCR (red arrow). (**B**) The microdeletion at 1q21.2 was verified by the CytoScan HD Array (red arrow). (**C**) Triplication of GJA8 was detected in the patient HCG889 by RT-qPCR (red arrow). (**D**) The microduplication was verified by the CytoScan HD Array (red arrow).

**Figure 2:**
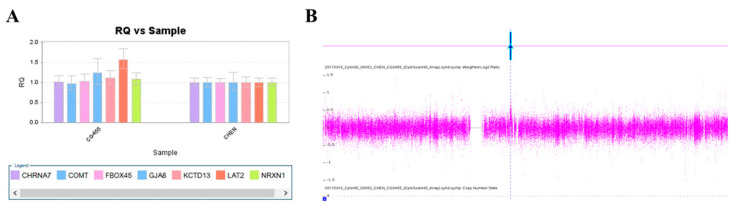
A microduplication at 7q11.23 was detected in this study. (**A**) Triplication of LAT2 in patient CG455 was detected by real-time quantitative PCR (red arrow). (**B**) The microduplication was verified by the CytoScan HD Array (red arrow).

**Figure 3:**
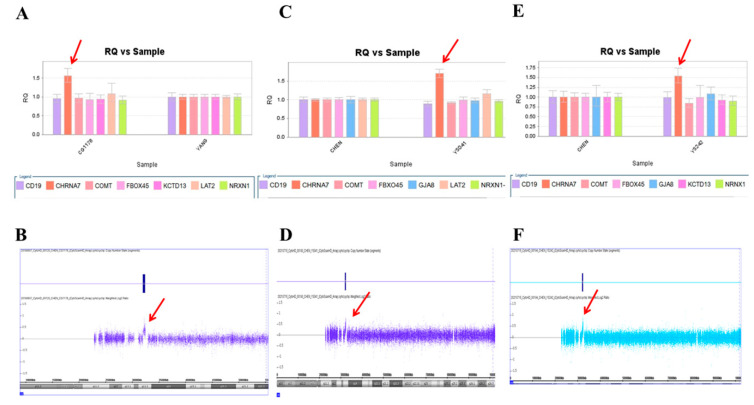
Three microduplications at 15q13.3 were detected in this study. (**A**) Triplication of CHRNA7 was detected in the patient CG1176 by real-time quantitative PCR (red arrow), which was verified by the CytoScan HD Array (red arrow) (**B**). (**C**) The patient YS041 also had triplication of CHRNA7 in the real-time quantitative PCR (red arrow), which was verified by the CytoScan HD Array (red arrow) (**D**). (**E**) A third patient, YS242, had triplication of CHRNA7 by real-time quantitative PCR (red arrow), which was verified by the CytoScan HD Array (red arrow) (**F**).

**Figure 4:**
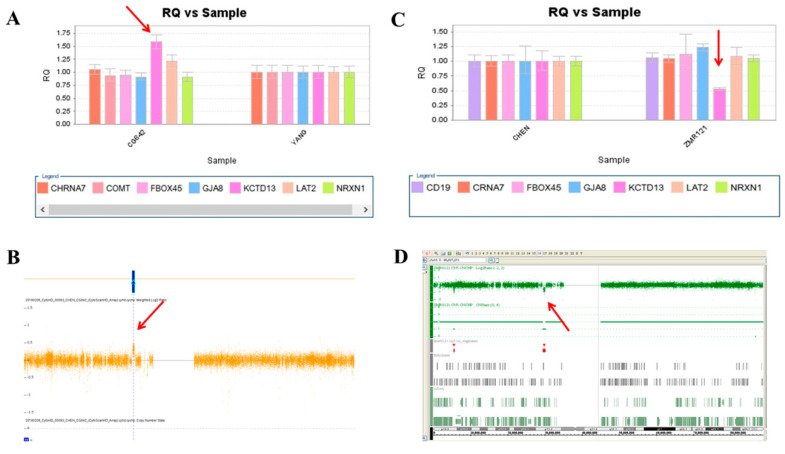
A microduplication and a microdeletion at 16p11.2 proximal were detected in this study. (**A**) Triple copies of KCTD13 were detected in patient CG842 by real-time quantitative PCR (red arrow), which was verified by the CytoScan HD Array (red arrow) (**B**). (**C**) A single copy of KCTD13 was detected in patient ZMR121 by real-time quantitative PCR (red arrow), which was verified by the Affymetrix Genome-Wide Human SNP Array 6.0 (red arrow) (**D**).

**Figure 5:**
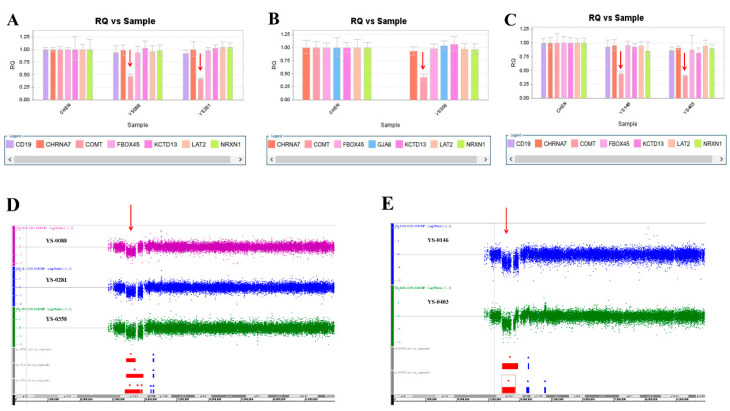
Five microdeletions at 22q11.2 were detected in this study. (**A**) The real-time quantitative PCR experiments revealed a single copy of COMT in patient YS088 and patient YS281 (red arrow), in patient YS358 (**B**) (red arrow), and in patient YS0146 and patient YS403 (**C**) (red arrow). These microdeletions were further defined by the Affymetrix Genome-Wide Human SNP Array 6.0 in patients YS088, YS281, and YS358 (**D**) (red arrow), and in patient YS146 and patient YS403 (**E**) (red arrow).

We used this panel to screen samples from 557 patients diagnosed with schizophrenia or schizoaffective disorders. We detected a total of thirteen positive cases from the screening test, including one patient with a microdeletion at 1q21.2 (Figure 1A), one patient with microduplication at 1q21.1 (Figure 1C), one patient with a microduplication at 7q11.2 (Figure 2A), three patients with microduplications at 15q13.3 (Figure 3A,C,E), one patient with a microduplication at proximal 16p11.2 (Figure 4A), one patient with a microdeletion at proximal 16p11.2 (Figure 4C), and five patients with microdeletions at 22q11.2 (Figure 5A–C). The CNV detection rate in this sample was 2.3%.

### 2.3. Confirmation by Chromosomal Microarray Analysis (CMA)

The positive cases detected from the screening experiments were subjected to genome-wide CNV analysis by either the Affymetrix Genome-Wide Human SNP Array 6.0 (Affymetrix, Santa Clara, CA, USA) or the CytoScan HD Array (Affymetrix, Santa Clara, CA, USA). The positive cases from the RT-qPCR screening test were confirmed to have a pathogenic CNV at the corresponding locus. Two patients positive at 1q21.1 carried a 1787 kb microdeletion and a 901 kb microduplication, respectively (Figure 1B,D). The patient positive at 7q11.2 had a 446 microduplication (Figure 2B). Three patients positive at 15q13.3 had a microduplication of 443 kb, 420 kb, and 435 kb, respectively (Figure 3B,D,F). Two patients positive at proximal 16p11.2 had a 586 kb microduplication (Figure 4B) and a 789 microdeletion (Figure 4D). Five patients were positive at 22q11.2 in the screening assay. Each one carried a microdeletion at 22q11.2, with sizes ranging from 1454 kb to 2696 kb (Figure 5D,E). The detailed genetic information on these CVNs is summarized in Table 2.

## 3. Discussion

Identifying the pathogenic CNVs in patients with psychiatric disorders has several important implications in clinical management. First, it may aid in making the etiological diagnosis for some patients and offer helpful information for genetic counseling of the affected patients and their families [24,25]. Second, it may help elucidate the pathophysiology of psychiatric disorders through the mechanistic study of pathogenic CNVs [26]. Third, it may provide targeted treatment and practical guidelines to treat patients with pathogenic CNVs in clinical settings [27,28,29,30].

This study developed an RT-qPCR test panel that simultaneously detected dose changes in selected genes at eight target loci where pathogenic CNVs recur in schizophrenia. We used this test to screen a sample of 557 patients and detected 13 positive cases. Further CMA analysis confirmed that each of the thirteen cases carried a pathogenic CNV at the corresponding target locus. The concordance rate between the RT-qPCR test and the CMA was 100%, indicating that the RT-qPCR test developed in this study is accurate and reliable. The cost of the RT-qPCR test is much cheaper than that of CMA. Moreover, it is less time-consuming and less technically challenging than CMA. Hence, the RT-qPCR test panel is more cost-effective than CMA in detecting the CNVs at the eight hot loci in clinical settings.

This study identified 13 patients carrying the pathogenic CNVs out of 557 patients; the detection rate was 2.3%. These CNVs occurred in five out of eight target loci, including five microdeletions at 22q11.2 (detection rate 0.9%) and three microduplications at 15q13.3 (detection rate 0.5%). CNVs at these two loci might be the patients’ most frequent recurrent CNVs. The other CNVs included one microduplication and one microdeletion at proximal 16p11.2 (detection rate 0.4%), one microdeletion and one microduplication at 1q21.1 (detection rate 0.4%), and one microduplication at 7q11.23 (detection rate 0.2%). This study did not detect positive cases carrying CNVs at 2p16.3 (NRXN1), 3q29, and distal 16p11.2. The differential detection rates for each of the eight target loci might be due to the different prevalence rates of CNVs at the eight loci in our patients and the small sample size used in this study. An increase in the sample size might help find patients carrying CNVs at these three loci.

The RT-qPCR panel in this study targeted the screening of CNVs at eight loci reported by the CNV working group of PGC [11]. The panel can be expanded to include more targeted loci. For example, the CNV working group of the PGC reported additional five loci as suggested risk CNVs loci of schizophrenia, including CNVs at Xq28 distal, 15q11.2, 9p24.3, 8q22.2, and 7p36.3 [11]. These risk loci can be included in this panel of RT-qPCR tests to screen more risk CNVs associated with schizophrenia. Furthermore, several studies reported that disease-associated CNV profiles differed in different populations and healthcare systems [31,32,33]. The target loci of the RT-qPCR test can be flexibly adjusted to meet the different needs of patients with different demographic backgrounds.

The RT-qPCR panel developed in this study was based on the study of schizophrenia-associated CNV by PGC [11]. However, studies show that schizophrenia shares some common heritability with other psychiatric conditions. The pathogenic CNVs at the eight target loci in this study are not limited to schizophrenia only; they are also pathogenic with other neurodevelopmental disorders and psychiatric conditions. For example, CNVs at 1q21.1 are associated with attention-deficit hyperactivity disorder, oppositional defiant disorder, anxiety disorder, autism spectrum disorder, tic disorder, intellectual disability, and depression [10,34,35,36]. The microdeletion of NRXN1 at 2p16.3 is associated with several neurodevelopmental disorders and intellectual disability, too [37,38]. Deletions at 3q29 are associated with intellectual disability, anxiety disorder, autism, and schizophrenia [39]. Duplications at 7q11.23 are associated with an increased risk for schizophrenia [40], while deletions at 7q11.23 lead to Williams syndrome, a neurodevelopmental disorder. Notably, deletions and duplications at 7q11.23 are associated with autism spectrum disorder [41,42,43,44]. CNVs at 15q13.3 are associated with developmental disorders, intellectual disability, attention-deficit hyperactivity disorder, autism spectrum disorder, childhood-onset schizophrenia, and depression in addition to schizophrenia [32,36,45,46]. CNVs at proximal 16p11.2 are also associated with neurodevelopmental disorder, intellectual disability, attention-deficit hyperactivity disorder, depression, and autism [34,36,47,48,49,50,51]. Finally, CNVs at 22q11.2 are associated with congenital heart disease, neurodevelopmental disorders, intellectual disability, autism spectrum disorders, and other neuropsychiatric conditions [7,48,52,53]. Together, these data indicate that clinical manifestations of CNVs at the eight loci in this study are not limited to schizophrenia; they have pleiotropic clinical effects. Hence, the RT-qPCR panel in this study can be reasonably used to screen CNVs in patients with developmental disorders and other psychiatric diagnoses.

This study has several limitations. First, the sample size of this study is small, which might affect the CNV detection rate at the eight target loci in this study. Future studies with increasing sample sizes are needed to address this issue. Second, the efficiency of RT-qPCR to detect CNVs is related to the slope of the standard curve of each primer pair. The failure to detect CNVs at three loci (2p16.3 (NRXN1), 3q29, and distal 16p11.2) in this study might also be due to the deviation of the slope values of three primer pairs from the ideal slope value (−3.1 to −3.6). We need to optimize the primer design for these three loci in future studies. Third, we did not perform the family analysis of patients who carried CNVs in this study. Hence, we could not determine the origin of these CNVs and offer genetic counseling for the patients and their families, which should be improved in future studies. Fourth, the recruitment of patients in this study was based on psychiatric diagnosis only. We did not collect detailed clinical information and psychometric assessment for the participants of this study, which limits our continuous study of the genotype–phenotype relationship. Future studies with more detailed clinical and psychometric assessments of participants should improve the quality of this research.

## 4. Materials and Methods

### 4.1. Subjects

We recruited patients fulfilling the diagnostic criteria of schizophrenia or schizoaffective disorder according to the DSM-5 (Diagnostic and Statistical Manual of Mental Disorder-5th edition) from two hospitals: the Department of Psychiatry, Chang Gung Memorial Hospital-Linkou, Taoyuan, Taiwan, and the Department of Psychiatry, Yuli Mental Health Research Center, Yuli Branch, Taipei Veterans General Hospital, Hualien, Taiwan. Patients with a history of head injury or other medical conditions associated with psychiatric disorders were excluded. The Institutional Review Board approved the study at Chang Gung Memorial Hospital with the approval number of 201901769B0 and the Institutional Review Board at Antai-Tian-Sheng Memorial Hospital with the approval number of 101,020, respectively. Informed consent was obtained from the patient or guardians after the full explanation of this study. Genetic DNA was extracted from venous blood using the Smart Genomic DNA Extraction kit (Intelligent Biomedicine, Taipei, Taiwan) or Gentra Puregene Blood kit following the manufacturer’s instructions (Qiagen, Hilden, Germany). All the experiments and informed consent procedures followed the relevant guidelines and regulations set by the research ethics committees of the two institutes. We obtained samples from of a total of 557 patients with schizophrenia spectrum disorders, including 290 males and 267 females. The mean age was 43 ± 15 years old.

### 4.2. Real-Time Quantitative PCR (RT-qPCR) Test of Target Loci

For detecting CNV at each target locus, we designed specific primer pairs to PCR amplify a small fragment of a gene encompassed by CNV at the target locus. For each PCR, we prepared a volume of 20 μL PCR mixture containing 100 ng genomic DNA, 1 μL each of forward and reverse primers (2.5 mM), and 10 μL of 2X SYBR Master Mix (Applied Biosystems, Foster City, CA, USA) RT-qPCR experiment was performed using the following conditions: after denaturing the mixtures at 95 °C for 10 min, 30 cycles of PCR were performed, of 95 °C for 30 s followed by 63 °C for one minute. After completing the PCR, we assessed the specificity of the amplicon using melting curve analysis. The experiments were implemented using the StepOnePlus machine according to the manufacturer’s instructions (Applied Biosystems, Foster City, CA, USA). PCR of each target gene was run in triplicate in each experiment. We generated a standard curve for each primer pair with serial dilutions of template DNA to assess the PCR efficiency for each primer set. We measured the slope of the standard curve of each primer set to assess the PCR efficiency [21]. The dose changes in the target gene were analyzed using the comparative ddCt method. In brief, the Ct of a reference gene (dCt) was subtracted from the Ct of the target gene, which was then normalized to a control subject (ddCt). Each gene could be used as the internal reference gene for other target genes in calculating the dose change. The relative fold change to a normal subject was determined as 2^−ddCt^. After optimizing the PCR conditions for each target locus, we assembled eight primer pairs as a panel and ran RT-PCR simultaneously under the same PCR conditions. Furthermore, to avoid pipetting errors and reduce variations within and between experiments, we used a liquid handling system to aliquot the reagents and samples. We used the optimized RT-qPCR test panel as the screening test to detect CNVs at the eight hot genetic loci reported by the CNV Analysis Group of the Psychiatric Genomics Consortium (PGC) [11].

### 4.3. Chromosomal Microarray Analysis (CMA)

We used two CNV analysis platforms to verify the positive cases detected from the RT-qPCR screening test. One was the Affymetrix Genome-Wide Human SNP Array 6.0 (Affymetrix, Santa Clara, CA, USA), performed by the National Genotyping Center (Academia Sinica, Taipei, Taiwan). The other was the CytoScan HD Array, performed by the Genomic Medicine Core Laboratory of Chang Gung Memorial Hospital-Linkou (Taoyuan, Taiwan). The experimental procedures followed the protocols provided by the manufacturers. Data from the Affymetrix Genome-Wide Human SNP Array 6.0 were analyzed and reported using the Affymetrix Genotyping Console software v.4.1 (Affymetrix, Santa Clara, CA, USA), whereas data from CytoScan HD Array were analyzed and reported using the software Chromosomal Analysis Suite Version 3.3.0.139 (r10838) (Affymetrix Inc., Santa Clara, CA, USA). The genomic coordinates of CNVs followed the human genome sequences version GRCh37/hg19.

## 5. Conclusions

We developed an RT-qPCR test panel targeting CNVs at eight specific loci. The panel has the flexibility to be expanded and customized to screen more specific recurrent CNVs in different situations. The RT-qPCR method is reliable and cost-effective and can be quickly adopted in clinical laboratories. Implementing the two-stage approach should facilitate the molecular diagnosis of schizophrenia and other neurodevelopmental disorders in some patients.

## Figures and Tables

**Table 1:** The genetic information of eight primer pairs used for real-time quantitative PCR in this study and the representative slope and regression coefficient (R^2^) of the standard curve generated by these primer pairs.

No	Locus	Target Gene	Sequences	Size (bp)	Slope	R^2^
1	1q21.1	GJA8	GJA8-F 5′-TTCAGGTGGGTGAGAAATGGGCG-3′ GJA8-R 5′-CAGAGGCGAATGTGGGAGATGGG-3′	249	−2.98	0.993
2	2p16.3	NRXN1	NRXN1-Exon 1-F 5′-CTGGCTCGAGCTGAGGGGAATAAC-3′ NRXN1-Exon 1-R 5′-CCACCACCCATCTTTCCCTGTGTA-3′	154	−3.03	0.999
3	3q29	FBXO45	FBXO45-F 5′-AGATTGGTTTCAGTGAGGGCCGC-3′ FBXO45-R 5′-ATTGTCCACCAGATTCCAGCCCC-3′	170	−2.547	0.991
4	7q11.23	LAT2	LAT2-F 5′-TCTTGGCCAGAGACCCCATTGC-3′ LAT2-R 5′-CTTCCTCCACCTCCGGCTTTGTC-3′	100	−3.12	0.997
5	15q13.3	CHRNA7	CHRNA7-F 5′-TCTCTCCTTAAGTGTCCCTGCAA-3′ CHRNA7-R 5′-CACCACGTCCATGATCTGCAGGA-3′	151	−3.124	0.999
6	distal 16p11.2	CD19	CD19-F 5′-GACTGGTGGCTGGAAGGTCTCAG-3′ CD19-R 5′-GATGGTTGTCAGACTGGCCAGAG-3′	173	−3.056	0.998
7	proximal 16p11.2	KCTD13	KCTD13-S 5′-CCCACTAGGTTGGGTGCTGATTGA-3′ KCTD13-AS 5′-AATCAGGCCCTGCACCAGGTAGTA-3‘	154	−3.452	0.998
8	22q11.2	COMT	COMT-S 5′-ATCCTGCAGCCCATCCACAACCTG-3′ COMT-AS 5′-ACGTTCATGGCCCACTCCTTCTGC-3′	289	−3.129	0.997

**Table 2:** The genetic information on CNVs confirmed in this study.

No	Locus	Case	CNV Type	Start	End	Size (kb)
1	1q21.1–1q21.2	LT49	Deletion	146,043,713	147,830,830	1787
2	1q21.1	HCG889	Duplication	146,498,298	147,399,659	901
3	7q11.23	CG455	Duplication	73,585,140	74,031,427	446
4	15q13.3	CG1176	Duplication	32,003,537	32,446,830	443
5	15q13.3	YS041	Duplication	32,024,190	32,444,196	420
6	15q13.3	YS242	Duplication	32,011,476	32,446,830	435
7	proximal 16p11.2	CG842	Duplication	29,591,078	30,177,224	586
8	proximal 16p11.2	ZMR121	Deletion	29,425,200	30,214,457	789
9	22q11.2	YS0088	Deletion	18,876,416	20,330,744	1454
10	22q11.2	YS0281	Deletion	18,876,416	21,572,202	2696
11	22q11.2	YS0358	Deletion	18,890,623	21,572,202	2682
12	22q11.2	YS0403	Deletion	18,876,416	21,034,809	2185
13	22q11.2	YS0146	Deletion	18,876,416	21,465,050	2589

## Data Availability

The raw data are available upon request of the corresponding author.
